# Early enteral nutrition in the critically ill: a single-centre study

**DOI:** 10.1186/cc10770

**Published:** 2012-03-20

**Authors:** A Gupta, E Rupert, R Sharma, D Mishra

**Affiliations:** 1 Rabindranath Tagore Hospital, Kolkata, India

## Introduction

Early enteral nutrition in critically ill patients has been established as a valuable addition to improve overall outcome and mortality. The recommendation is to initiate feeding within 24 to 48 hours of admission and to meet the calorie goal within the next 48 to 72 hours. The purpose of this study was to find out whether these guidelines are followed in the ICU as per the new protocol.

## Methods

This is a prospective observational study done in a 32-bed mixed medical and surgical ICU over the period from March 2011 to August 2011. Consecutively, 575 patients admitted to this ICU were followed up. Nineteen patients were excluded from the study where enteral nutrition could not be commenced within 48 hours due to various reasons. The remaining 556 patients' data were analyzed. Data were collected by interviewing the doctors and nurses as well as reviewing medical notes and all ICU charts.

## Results

In the 556 study patients, early enteral feeding was started in 379 patients (68.16%). Out of 379 patients, 100% calorie requirements were met only in 43 patients (7.73%). For the remaining study patients, more than 40%, 50% and 60% calorie goals were achieved in 115 (30.34%), 128 (33.77) and 93 (24.53%) patients respectively.

## Conclusion

The initiation of early enteral feeding is still far off for a significant proportion of the ICU population despite evidence-based definite recommendations to improve ICU outcome. The calorie goal achievements were also very suboptimal. This important but still neglected nutritional therapy must be carefully looked at and implemented in all ICUs.
